# 3D‐Nanostructured ITO Enables High‐Loading Prussian Blue Cathodes for Zn‐Based Electrochromic Smart Windows

**DOI:** 10.1002/smtd.71123

**Published:** 2026-10-15

**Authors:** Renald Sangarane, Hamid Palamadathil Kannattil, Kenneth D. Harris, Benoît Limoges, Véronique Balland

**Affiliations:** ^1^ Université Paris Cité, CNRS UMR 7086 ITODYS Paris France; ^2^ National Research Council Canada Quantum and Nanotechnologies Research Centre Edmonton Alberta Canada; ^3^ Department of Mechanical Engineering University of Alberta Edmonton Alberta Canada

**Keywords:** aqueous electrochromic battery, energy‐storage smart windows, nanostructured electrode, zinc electroplating

## Abstract

Prussian Blue (PB) is an attractive electrochromic cathode material for aqueous electrochromic energy‐storage smart windows, but its performance remains limited by the low areal capacity of thin‐coatings deposited on planar transparent conductive oxides. Here, we overcome this limitation by combining galvanostatic electrodeposition with 3D‐nanostructured ITO electrodes, enabling conformal growth of PB with high‐loadings, while maintaining mechanical stability and fast electrochemical response. The resulting electrodes sustain charge densities up to 100 mC/cm^2^ at relatively high rates. When integrated into Zn/PB smart window devices, they enable fast and homogeneous coloration/bleaching with enhanced optical contrast and charge storage capacity, while also exhibiting high cyclability. Device configurations based on reversible Zn electrodeposition onto a transparent ITO counter electrode enable improved control over optical contrast and electric field distribution, while a three‐electrode architecture incorporating a sacrificial Zn anode provides a route toward in situ regeneration of the electrochromic cathode and extended device lifetime.

## Graphical abstract

Transparent nanostructured ITO electrodes prepared by Glancing Angle Deposition enable conformal Prussian Blue electrodeposition, boosting surface capacity and rate capability. Coupled with reversible zinc plating on a zinc frame and ITO counter electrode, the energy‐storage smart window achieves fast optical modulation and improved charge‐storage performance, while an in‐situ rejuvenation mode enhances cyclability.

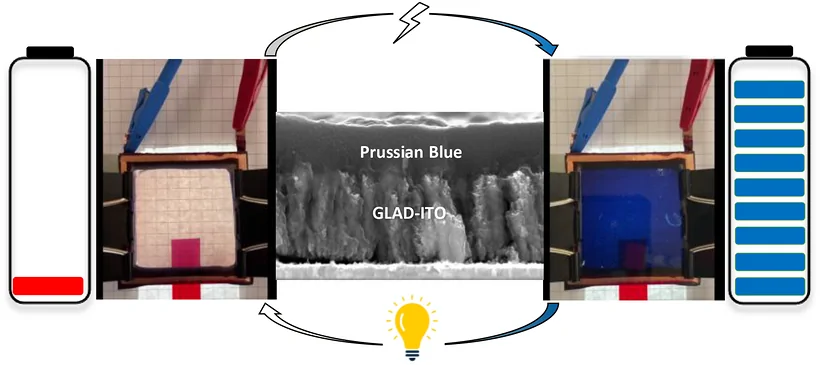

## 1 Introduction

With accelerating climate change, developing energy‐efficient technologies using abundant, nontoxic, and sustainable materials is essential for carbon emission reduction and climate‐neutral energy systems. Prussian blue (PB), a compound with remarkable properties and a rich history, stands out as an example of how abundant and eco‐friendly constituents can drive advancements in various fields. Being composed of iron(III) hexacyanoferrate(II), Prussian Blue is a deep blue synthetic pigment, first synthesized in 1706 [1]. Beyond its historical role in art and decoration, PB has emerged as a versatile metal oxide framework. Its good chemical stability, high ion‐exchange capacity and photo/redox active constitutive iron ions make it an interesting material for applications in medicine [2, 3], environmental remediation [4], energy storage [5, 6], and electrochromism [7]. This highlights the broad potential of Prussian Blue‐based technologies, ranging from electrochemical sensors and batteries to smart windows [8]. Interestingly, Prussian Blue also provides the opportunity to achieve innovative multifunctional electrochromic devices [9].

The optical properties of Prussian Blue stem from an intervalence charge‐transfer transition between low‐spin Fe^2^^+^ and high‐spin Fe^3^^+^ ions, which alternate at the corners of the cubic lattice and are interconnected by cyanide bridges. Accordingly, the electrochromic behavior of the mixed‐valence Prussian Blue arises from electrochemical switching between Prussian Blue (PB, Fe^3+^/Fe^2+^) and Prussian White (PW, Fe^2+^/Fe^2+^) upon reduction, or Prussian Yellow (PY, Fe^3+^/Fe^3+^) upon oxidation. Most electrochromic applications of Prussian Blue exploit the PB/PW redox transition, owing to the low stability of Prussian Yellow and the need to maximize the color change. Therefore, PB is generally referred to as an anodic electrochromic material, that is, coloring upon oxidation and bleaching upon reduction [7]. This PB/P redox reaction has been shown to be highly reversible and stable in mild acidic aqueous electrolytes containing K^+^ cations as charge carrier [10, 11, 12], a process associated with the reversible insertion of K^+^ in the PB structure during reduction to locally compensate for the negative charge injected into the material [13]. The reversible insertion of cations in PB is facilitated by its open lattice structure, exhibiting large interstitial sites (4.6 Å) and channels (3.2 Å) [14]. Consequently, PB has also been shown to reversibly insert many cations beyond K^+^ [15, 16, 17], making it a versatile cation‐insertion material. This versatility, combined with suitable redox potentials and gravimetric capacities, underpins the current strong interest in PB and its derivatives as cathode materials for aqueous batteries, beyond Li‐ion devices [6, 18].

In this context, Prussian Blue appears as an attractive electrochromic cathode material for the development of electrochromic energy storage devices [19, 20, 21]. Unlike traditional electrochromic devices, which consume energy during coloration and bleaching, electrochromic energy storage devices achieve color/bleach transitions while reversibly storing energy. This dual‐purpose functionality offers an efficient solution for a wide range of applications, from colorimetric state‐of‐charge monitoring in batteries to energy storage smart windows (ESSWs). In the specific case of ESSWs, stringent criteria for eco‐sustainability, safety, durability and cost‐effectiveness must be met, and to achieve this, priority should be given to systems that operate in mild aqueous environments and utilize abundant, nontoxic materials. Furthermore, the performance of such systems must be assessed not only in terms of optical criteria, such as the range and rate of transmittance modulation, but also in terms of key energy performance metrics, including energy density and efficiency [22]. For all these reasons, combining an electrochromic Prussian Blue cathode with a metallic zinc anode—a strategy leveraging zinc's abundance and low reversible electrodeposition potential—emerges as a promising approach for ESSWs [23, 24, 25, 26, 27, 28, 29, 30]. In these devices, energy performance currently remains limited by a surface capacity below 80 mAh/m^2^ due to the low loading of Prussian Blue at the cathode, which constrains the overall charge storage capacity. For the same reason, these devices fail to achieve low transmittance values (<10%) in the visible range after charging. To overcome this limitation, it is essential to achieve reversible cycling of a larger amount of PB while maintaining a color‐switching time compatible with the targeted ESSW application. This is a challenging task requiring careful design of the high‐loading electrochromic PB electrode, which is the topic of the present study.

Electrochromic Prussian Blue electrodes are commonly prepared on transparent conductive substrates by a diverse range of wet processes, including hydrothermal synthesis, electrodeposition, and more recently, slot‐die coating or inkjet printing [7]. Among them, galvanostatic electrodeposition is a simple and cost‐effective method that allows excellent control over the deposited film by adjusting the current and time, and is easily adaptable to a range of conductive substrates of complex morphology. In the first demonstration by Itaya, SnO_2_ electrodes were coated with PB through electrochemical reduction of an acidic aqueous solution containing a mixture of FeCl_3_ and K_3_[Fe(CN)_6_] [15]. Since then, this method has been implemented on various transparent conductive substrates, mainly planar ITO and FTO electrodes [31, 32], but also, more recently, on an FTO electrode covered by a mesoporous semi‐conductive TiO_2_ thin film [33]. It was also the preferred methodology for preparing most of the Zn/PB ESSWs reported so far [23, 26, 27, 28]. However, in these reports, the charge passed during the electrodeposition process was typically below 20 mC/cm^2^ (corresponding to a film thickness of ca. 350 nm) in order to ensure good adhesion of the PB coating on the transparent conductive substrate [23]. This imposes an intrinsic limitation on the charge storage capacity of the Zn/PB ESSWs. To overcome this technological barrier, our strategy relies on combining a transparent mesoporous electrode with high specific surface area and a galvanostatic electrodedeposition process enabling the conformal growth of a Prussian Blue film throughout the electrode porosity. This innovative approach is designed to enable the deposition of a large amount of PB while maintaining good adhesion and homogeneity, as well as a fast electrochromic response.

To this end, we leverage 3D‐nanostructured and transparent ITO electrodes prepared by glancing angle deposition (GLAD) [34]. This one‐step vapor deposition method allows the growth of a conductive mesoporous film whose porosity, transparency, and thickness can be easily adjusted. GLAD‐ITO electrodes were previously used by us to achieve reversible electrodeposition of MnO_2_ and to develop an innovative Zn/MnO_2_ ESSW [35, 36]. In the present study, we demonstrate their potential to overcome the areal capacity limitations of PB electrochromic films. We further explore their integration into ESSW devices as an electrochromic cathode, paired with an anode exploiting the reversible electrodeposition of zinc either directly onto a metallic zinc frame or, more ambitiously, onto a commercial planar transparent ITO electrode arranged face‐to‐face, for a dual‐tinting ESSW.

## 2 Results and Discussion

### 2.1 Nanostructured Electrodes

Nanostructured GLAD‐ITO thin films were deposited on commercial ITO‐glass substrates according to the literature (see Experimental Section ‐ Supporting Information for details) [34]. The deposition angle was set to 85° to ensure a large porosity of 0.66 [34], and the film thickness was 950 ± 50 nm, as measured by cross‐sectional scanning electron microscopy (SEM) (Figure 1C). The SEM images shows a well‐organized array of nanocolumns grown perpendicularly to the planar ITO substrate. The electroactive surface area of the nanostructured GLAD‐ITO electrode was estimated to be enhanced by a factor 16, based on a comparison of its electrical double‐layer capacitance (determined by cyclic voltammetry and normalized to the geometric electrode surface area) with that of a commercial planar ITO electrode (Figure S1).

**FIGURE 1:**
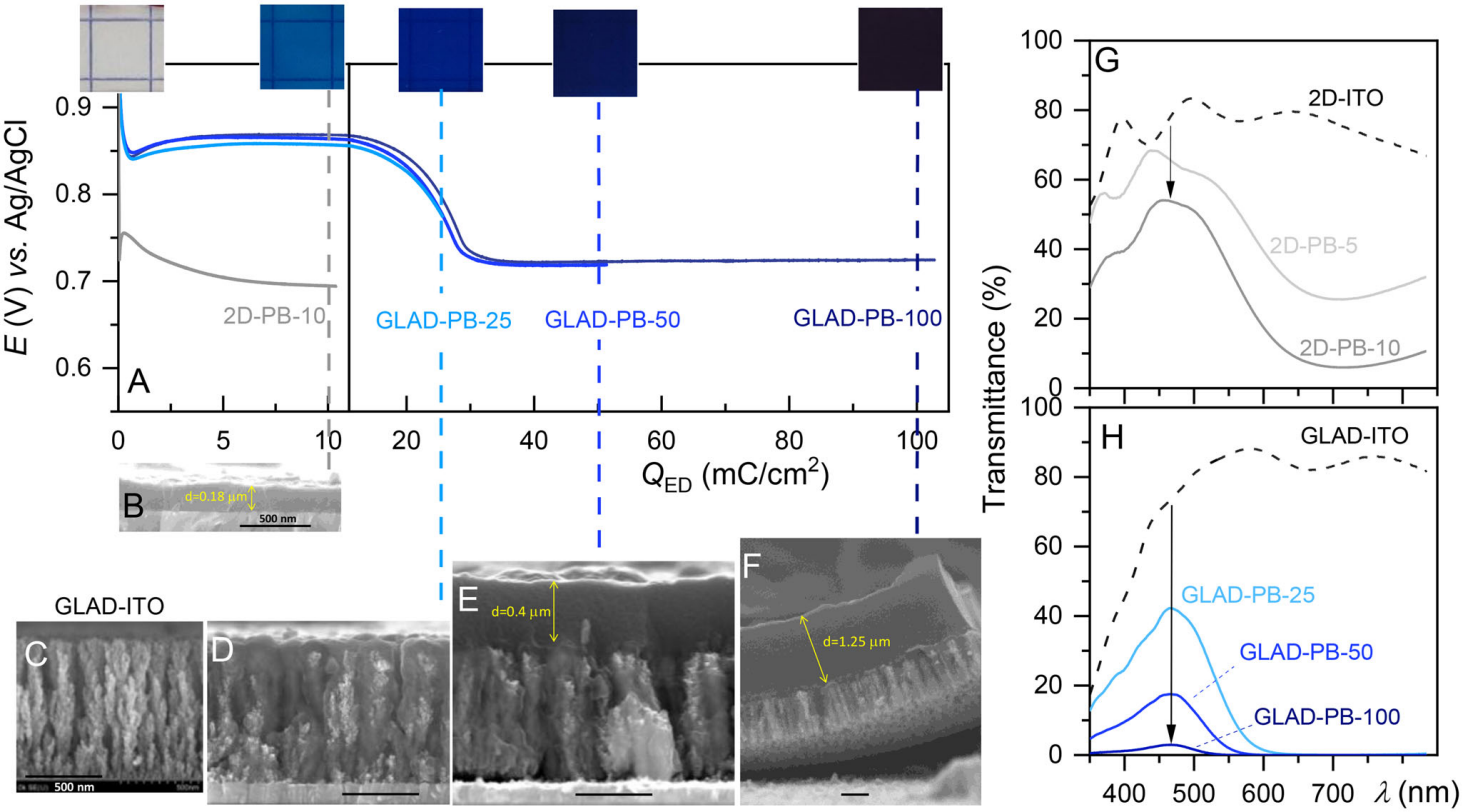
(A) Galvanostatic electrodeposition of Prussian Blue on (gray curve) 2D‐ITO and (blue curves) GLAD‐ITO for a total charge passed of 10 mC/cm^2^ on 2D‐ITO and (from light to dark blue) 25, 50, and 100 mC/cm^2^ on GLAD‐ITO. (B–F) Cross‐sectional SEM images of the modified electrodes, along with an image of (C) the pristine GLAD‐ITO electrode. Black scale bars correspond to 500 nm. (G, H) UV‐visible transmission spectra of the (G) 2D‐ITO and (H) GLAD‐ITO electrodes after removal from the deposition solution and drying. Spectra from unmodified electrodes are shown as dashed lines, and those from modified electrodes are shown as solid lines.

### 2.2 Electrodeposition

PB‐GLAD‐ITO electrodes were prepared by galvanostatic electrodeposition, by applying a constant reductive current of ‐50 µA/cm^2^ (normalized to the geometric electrode surface area) at GLAD‐ITO electrodes (with sizes ranging between 0.9 × 2.5 cm^2^ and 5 × 5 cm^2^, see Figure S2) soaked in an aqueous electrolyte containing equimolar amounts of FeCl_3_ and K_3_Fe(CN)_6_, along with KCl as supporting electrolyte (see Experimental Section ‐ Supporting Information for details) [15]. The same protocol was also used to modify commercial flat ITO electrodes (2D‐ITO). The deposition times were chosen to set the amount of charge passed (*Q*_ED_) to values ranging from 25 to 100 mC/cm^2^ on GLAD‐ITO and 5 to 10 mC/cm^2^ on 2D‐ITO electrodes. All modified electrodes exhibited a highly homogeneous blue coloration characteristic of PB, which grew more intense as the deposition time was increased (see Figures 1 and S2). The evolution of the electrode potential upon electrodeposition, shown in Figure 1A, differed significantly between flat and nanostructured electrodes. At 2D‐ITO, the electrode potential steadily decreases from 0.75 to 0.7 V over 200 s. *On contrario*, after an initial sharp decrease, the potential of the GLAD‐ITO electrode stabilizes over ca. 200 s at a high value of 0.85 V, before suddenly decreasing up to a constant value of 0.72 V after 600 s. After deposition, modified electrodes were carefully rinsed and heated at 60°C overnight prior to further characterization and use. For clarity, modified electrodes were labelled 2D‐PB‐*Q*_ED_ and GLAD‐PB‐*Q*_ED_, with *Q*_ED_ specifying the charge value passed during the electrodeposition process (e.g., GLAD‐PB‐25).

### 2.3 Characterization of the Electrodeposited Film

The morphology of the modified electrodes was characterized by SEM, with top‐view images confirming the high homogeneity of the electrodeposited film (Figure S3). It is important to note that the macroscopic cracks and delamination defects observed in these SEM images (Figure S3 and Figure 1E,F) occur during the drying process. The film's chemical composition was further analyzed by EDX (Figures S3 and Table S1), which confirmed the presence of Fe, C and N in the deposited film. The C/N ratio is close to unity in all samples, while the potassium content remains low, confirming that the “insoluble” form of PB is the main component of the electrodeposited film, as commonly reported for galvanostatic electrodeposition at such low reducing current densities (typically ‐10 to ‐50 µA/cm^2^) [15, 32, 37].

Cross‐sectional SEM images (Figure 1) show that PB first deposits conformally within the porosity of the GLAD film (Figure 1D), coating the ITO nanocolumns throughout. As the charge passed increases further, the PB material extends beyond the porous GLAD‐ITO structure and forms a dense overlayer (Figure 1E,F). The transition between these two electrodeposition regimes—inside and outside the GLAD film porosity—is highly reproducible (see Figure S4) and coincides with a sudden shift in electrode potential after ∼25 mC/cm^2^ has been passed, toward a less favorable (i.e., a less positive) reduction potential once PB growth extends beyond the GLAD film porosity.

To better understand the origin of this potential shift between the two regimes observed on GLAD‐ITO electrodes, we examined how the reduction current density used for galvanostatic PB deposition affects the electrode potential on a 2D electrode (Figure S4). The electrode potential shifted to less positive values as the applied current density increased, consistent with previous observations by Itaya [15], and indicative of a kinetically controlled reductive electrodeposition process. Notably, on the 2D electrode, the deposition potential at ‐50 µA/cm^2^ matches the value observed on the GLAD electrode once PB growth extends beyond the porosity of the nanostructured GLAD film. In contrast, the potential associated to conformal PB deposition within the GLAD electrode porosity is close to that obtained on a 2D electrode at a lower current density of ‐6 µA/cm^2^. This suggests that PB electrodeposition within the porosity of the GLAD film is kinetically more favorable than on the 2D electrode or on top of the GLAD film, and that once deposition occurs outside the porosity, the GLAD electrode behaves like a flat electrode. We can therefore conclude that, from approximately 25–30 mC/cm^2^ onward, PB deposited on GLAD electrodes forms an overlayer similar to that deposited on 2D electrodes. This conclusion is further supported by the thickness *d* of the PB overlayer (Figure S5b), measured from SEM cross‐sections for both the 2D electrode and the regions of the GLAD‐ITO electrode extending beyond the porosity: both show a similar linear dependence of thickness on the charge passed during electrodeposition.

The transmittance spectra of the dried modified electrodes are reported in Figure 1G,H, showing the typical feature of PB with a transmittance minimum at *λ* ∼710 nm. As expected, transmittance decreases as the charge passed during electrodeposition increases, reaching for the modified GLAD electrodes (Q_ED_ ≥ 25 mC/cm^2^) values below 0.1% at 710 nm. Given this very low transmittance, corresponding to a saturated absorbance value, it was not possible to quantify the amount of PB using the extinction coefficient value available in the literature [15]. This was instead achieved by X‐ray fluorescence spectroscopy (XRF), where the amount of electrodeposited iron was observed to increase linearly with the charge passed during electrodeposition (Figure S5), on both planar and GLAD electrodes, independently of the deposition regime described above. The slope of the linear regression was 1.13 µg/mC, corresponding to a ratio of 2 Fe electrodeposited per electron passed—only slightly lower than the theoretical value of 2.3 expected for the electrodeposition of the “insoluble” mixed‐valence PB state, $Fe_{4}^{III}{\left[Fe^{II}{\left(CN\right)}_{6}\right]}_{3},\;xH_{2}O,$ according to Reaction 1:

(1) $$3{\left[Fe{\left(CN\right)}_{6}\right]}^{3-}+4Fe^{3+}+3e^{-}\;\to \;\;Fe_{4}^{III}{\left[Fe^{II}{\left(CN\right)}_{6}\right]}_{3}$$

This insoluble state is described as a defect‐rich lattice, with 25% hexacyanoferrate vacancies and two kinds of water molecules [38]. The Fe/e^−^ ratio translates into an average faradaic efficiency of ca. 87% for the electrodeposition process, suggesting the possible concomitant formation of a small amount of poorly or non‐adherent PB. Note that the data point corresponding to the 25 mC/cm^2^ deposition lies slightly above the linear regression curve, which otherwise shows low dispersion (Figure S5). This likely reflects a higher electrodeposition efficiency of PB when it occurs within the porosity of the GLAD‐ITO film, compared to when it subsequently occurs above the GLAD structure.

From the slope values of Figure S5 linking, on one hand, the PB thickness *d* to *Q*_ED_, and, on the other hand, the amount of electrodeposited Fe as a function of *Q*_ED_, one can estimate the density of PB electrodeposited on both the 2D‐ITO electrode and on top of the GLAD‐ITO electrode structure. An average value of ∼0.63 g_Fe_/cm^3^ is obtained. Considering a stoichiometry of 7 Fe ions per PB unit, this value translates into ∼1.6 × 10^−3^ mol_PB_/cm^3^, which is consistent to the value of 1.58 × 10^−3^ mol_PB_/cm^3^ tabulated for crystalline insoluble PB (PDF Card No. 01‐073‐0689; density 1.76 g/cm^3^; molecular mass = 1111 g/mol) [38]. This calculation further confirms that our experimental data are fully consistent with the electrodeposition of a dense, insoluble PB coating.

The electrodeposited film was further characterized by X‐Ray diffraction and Raman spectroscopy (Figure S6). The Raman spectra of the 2D‐PB‐10, GLAD‐PB‐25, and GLAD‐PB‐50 are very similar and typical of the mixed‐valence PB [39, 40]. They all exhibit numerous peaks in the 200–650 cm^−1^ region, characteristic of the Fe‐C (541 cm^−1^) and Fe–N (477 cm^−1^) stretching modes and Fe‐C≡N‐Fe (365 cm^−1^) bending mode [39], as well as two intense peaks at 2090 and 2150 cm^−1^, associated with the C≡N stretching modes [40]. The XRD patterns of the GLAD‐PB‐25 and GLAD‐PB‐50 electrodes are very similar to one another, showing a series of peaks characteristic of the face‐centered‐cubic lattice (PDF Card No. 01‐073‐0689). Note that some peaks overlap with those of the ITO substrate, notably those located at 35.6° and 51.1°.

### 2.4 Electrochemical Analysis

After electrodeposition, the modified electrodes were systematically preconditioned through a series of 20 cyclic voltammograms in a three‐electrode cell containing a Pt counter electrode, an Ag/AgCl reference electrode, and a deaerated 3 M KCl electrolyte at pH = 3 [12, 41]. During these cycles (scanning from 0.6 to ‐0.2 V vs. Ag/AgCl at 5 mV/s), the mixed‐valence PB form is reversibly reduced to the PW state, which is associated with the reversible insertion of K^+^ cations, according to the following Reaction 2:

(2) $$Fe_{4}^{III}{\left[Fe^{II}{\left(CN\right)}_{6}\right]}_{3}+4K^{+}+4e^{-}\;⇌\;\;K_{4}Fe_{4}^{II}{\left[Fe^{II}{\left(CN\right)}_{6}\right]}_{3}$$

In the initial cycles, the peaks gradually narrow before stabilizing (see Figure S7). It has been proposed that this “stabilization process” is associated with the partial/total conversion of insoluble PB into soluble PB, that is, $KFe^{III}\left[Fe^{II}{\left(CN\right)}_{6}\right]$, upon loss of high‐spin Fe^III^ and incorporation of K^+^ cations [12]. However, this conversion, associated with the loss of ¼ high spin Fe^III^ ions, is far from being well‐understood and has recently been critically discussed [42]. In the present case, the charge reversibly cycled (Figure S7E), the chemical composition of the electrode (see Table S1), and the amount of Fe at the electrode (stars in Figure S5A) do not show any significant change after electrode stabilization, and no significant changes in film morphology were observed during this preconditioning step (Figure S8). We thus assume that the stabilization process has little effect on the film composition, which remains insoluble PB.

The electrodes were next characterized by cyclic voltammetry (CV) over a range of different scan rates in the aqueous KCl electrolyte (adjusted to pH 3). The CVs recorded for the different modified electrodes are shown in Figures 2A and S8. For all electrodes, the charge passed during the reductive scan is reported in Figure 2B, while the dependences of the peak potential and charge passed on the scan rate are plotted in Figure 2C, D, respectively.

**FIGURE 2:**
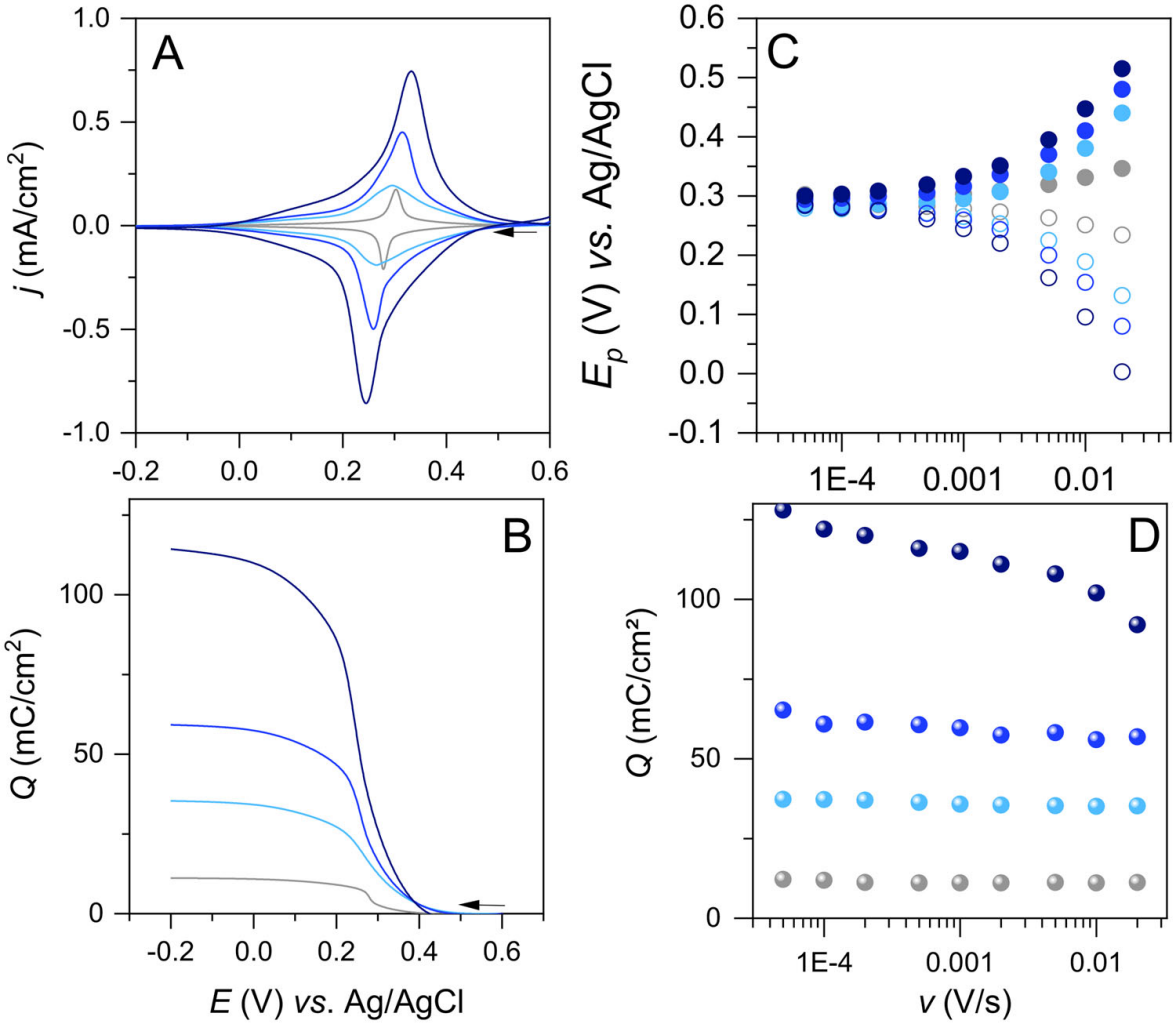
(A) Cyclic voltammograms recorded at 1 mV/s at (gray) 2D‐PB‐10, (light blue) GLAD‐PB‐25, (middle blue) GLAD‐PB‐50, and (dark blue) GLAD‐PB‐100 electrodes, soaked in a 3 M KCl aqueous electrolyte at pH 3. (B) Charge associated with the cathodic scan, recorded at 1 mV/s, with same color code as in A. (C) Peak potentials and (D) charge passed during the cathodic scan plotted as a function of scan rate. Filled and open circles correspond to the oxidative and reductive peaks, respectively.

The CVs of the electrodes exhibit a similar overall shape, characterized by a well‐defined pair of faradaic peaks associated with the reversible insertion of K^+^ and centered at an *E*_1/2_ value of 0.29 V versus Ag/AgCl, except for the GLAD‐PB‐25 electrode, whose CV displays a much broader envelope and an *E*_1/2_ value slightly downshifted to 0.28 V. Still, we note that the hysteresis between the reduction and oxidation peaks increases (from 24 to 90 mV) as the amount of electrodeposited PB increases (from 10 to 100 mC/cm^2^). This hysteresis appears to be strongly dependent on the scan rate, as shown in the trumpet plot (Figure 2C). Upon decreasing the scan rate to 0.05 mV/s, all faradaic peaks become much sharper (see Figure S9)—especially the cathodic peak, for which minimal full‐width‐half‐maximum (fwhm) values range between 10 and 20 mV, except for the GLAD‐PB‐25 electrode (fwhm = 110 mV). At low scan rates, the peak hysteresis also decreases below 25 mV for all electrodes.

The charge passed during the reduction scan corresponds to 11, 35, 60, and 114 mC/cm^2^, for the 2D‐PB‐10, GLAD‐PB‐25, GLAD‐PB‐50 and GLAD‐PB‐100 electrodes, respectively. We note that these values are systematically higher than the charge passed during the electrodeposition process. This is fully consistent with the previous observation that the faradaic efficiency of the electrodeposition process remains below 100%, and also with the fact that the reversible PB‐to‐PW cycling involves 4e^−^ per PB unit (Reaction 2) whereas PB electrodeposition is a 3e^−^ per PB unit reductive process (Reaction 1). Taking this stoichiometry into account, along with the faradaic efficiency of 87% as previously determined, *Q*_ED_ = 50 mC/cm^2^ is expected to translate into a final capacity of 58 mC/cm^2^, very close to the experimental value obtained here. This confirms that the entire deposited PB material is electroactive and also that it can be reversibly cycled between the PB and the PW states. The charge reversibly passed during cycling remains constant and independent of the scan rate (Figure 2D), except for the GLAD‐PB‐100 electrode. This constant charge attests to the good stability of the modified electrodes upon cycling, even at an extremely low scan rate of 0.05 mV/s for which complete CV recording requires 10 hrs. It also indicates a diffusion‐free, finite process, where the overall redox‐active centers in the PB layer are fully and reversibly converted within the time‐scale of the experiment, and this holds true even at scan rate as high as 20 mV/s.

The sharp faradaic peaks in CV are a common feature of electrodeposited PB coatings [13, 15], and a thermodynamic model assuming a regular solution behavior with a positive enthalpy of mixing was previously proposed by McCargar and Neff to account for this sharpness [13]. This nonideal shape of the faradaic peaks can also be thermodynamically described using a Frumkin isotherm, as initially proposed by Levi and Aurbach to account for interactions between insertion sites within the host lattice [43]. Under these conditions, the equilibrium electrode potential of the faradaic process coupled to cation insertion can predicted with the following modified Nernst equation:

(3) $$E=E_{PB/PW}^{0}+\frac{RT}{F}\ln a_{K_{aq}^{+}}+\frac{RT}{F}g\left(\theta -0.5\right)+\frac{RT}{F}\ln\frac{\theta }{1-\theta }$$

where $E_{PB/PW}^{0}$ is the standard potential associated with the PB/PW material, defined at half charge (i.e., θ = 0.5), $a_{K_{aq}^{+}}$ is the activity of the inserting cation (here K^+^) present in the electrolyte, θ is the state‐of‐charge (i.e., the mole fraction of the oxidized PB state) of the material, *g* is the phenomenological Frumkin parameter characterizing local repulsive/attractive interactions, *T* is the temperature, *R* = 8.314 J/mol/K and *F* = 96485 C/mol. The theoretical CV of the 2D‐PB‐10 electrode under conditions close to the thermodynamic equilibrium, that is, at *v* = 0.2 mV/s, could be satisfactorily reproduced from the time derivative of the Nernst Equation 3 and using the following set of data: $E_{PB/PW}^{0}$ = 0.274 V versus Ag/AgCl and *g* = ‐3 (see Figure S10A and the associated comment). The *g* value of ‐3 indicates the occurrence of attracting interactions within the host lattice, also characteristic of a phase separation leading to the coexistence of K^+^‐poor oxidized and K^+^‐rich reduced phases [43].

We currently lack a clear explanation for the significantly broader peaks observed with the GLAD‐PB‐25 electrode. This behavior appears to be highly specific to PB deposited within the nanostructured GLAD film, as both GLAD‐PB‐50 and GLAD‐PB‐100 electrodes recover sharp faradaic peaks. A more detailed analysis of the cyclic voltammograms of these higher‐loaded GLAD electrodes nevertheless suggests a distinct contribution from the PB located within the GLAD porous structure compared to the one located outside. To investigate this, we attempted to reconstruct the CV of the GLAD‐PB‐50 electrode by combining the response of the GLAD‐PB‐25 electrode—representing PB confined within the porosity of the GLAD electrode—with that of the 2D‐PB‐10 electrode, mimicking the dense film of PB deposited outside the porosity of the GLAD film. The reconstruction was based on the total charge passed (see Figure 2D). Accordingly, the CV of the GLAD‐PB‐25 electrode was combined with twice that of the 2D‐PB‐10 electrode. The resulting reconstructed CV (Figure S10B, red dashed line) shows excellent agreement with the experimental data, nearly overlapping with it. It should be noted that this approach is only valid at low scan rates, that is, under near‐equilibrium conditions. Indeed, the scan‐rate dependence of peak potential hysteresis strongly depends on the total amount of electrodeposited PB and is particularly pronounced for GLAD‐ITO electrodes (Figure 2C). The GLAD‐PB‐100 CV could also be satisfactorily reconstructed by combining the CV of the GLAD‐PB‐25 electrode with six times that of the 2D‐PB‐10 electrode (Figure S10C), whereas a poorer agreement was obtained by simply doubling the intensity of the GLAD‐PB‐50 CV. The three‐fold increase in the contribution of the 2D‐PB‐10 CV required to reconstruct the GLAD‐PB‐100 electrode, compared to the GLAD‐PB‐50 electrode, is fully in line with the increase in thickness of the dense film grown over the GLAD porosity (Figure 1E,F). Overall, this analysis supports the view that GLAD‐PB‐50 and GLAD‐PB‐100 electrodes consist of two distinct PB compartments having each a different electrochemical signature: (i) one associated to the PB material deposited within the porosity, leading to a broad voltammetric envelope, and (ii) one related to the dense PB layer formed outside the porosity, responsible for the sharp faradaic peaks.

### 2.5 Electrolyte Optimization

With the aim of coupling PB electrodes with zinc anodes in a smart window device, we first investigated the electrochemical behavior of a modified 2D‐PB‐10 electrode in a series of electrolytes with similar ionic strengths (3–3.5 M), containing either KCl (3 M), zinc acetate (1 M), or a mixture of both salts (2 M KCl + 0.5 M Zn(CH_3_COO)_2_). As shown in Figure S11, the capacity of the PB electrode remains extremely low in the electrolyte containing only zinc acetate. In contrast, the galvanostatic curves and the reversibly cycled capacity in KCl and the mixed KCl‐Zn(CH_3_COO)_2_ electrolyte are much higher than in the zinc acetate electrolyte, and remains close to each other. These observations agree with a charge storage mechanism relying exclusively on the reversible insertion of K^+^ in PB, thus without contribution from Zn^2+^ insertion, in contrast to what has sometimes been reported [44]. The good cyclability of the modified electrodes in the dual‐salt electrolyte was further confirmed at a GLAD‐PB‐50 electrode, which exhibited an initial capacity of 50 mC/cm^2^ at 1 mA/cm^2^, and a capacity retention of 57% after 1000 cycles. Further improvement of electrode cyclability, up to 70% under identical cycling conditions, was achieved upon addition of PEG‐200 (10% vol) and glycerol (50% vol). These additives were selected due to their beneficial effect on the reversible electrodeposition of zinc on ITO electrodes [45]. In Zinc‐based aqueous batteries, PEG derivatives are commonly use as additives to tune the electrode/electrolyte interface, while glycerol to improve reversible zinc electrodeposition in symmetric Zn cells, by limiting competitive hydrogen evolution and finally dendrite growth [46]. It was also shown that glycerol additive (up to 50% weight) allows to significantly inhibit PBA dissolution, while still allowing electrochemical cycling [47]. The addition of PEG‐200 and glycerol to the base aqueous electrolyte (2 M KCl, 0.5 M Zn(OAc)_2_) leads to a marked decrease in ionic conductivity, from 142 mS/cm for the base electrolyte to 89 mS/cm upon addition of PEG‐200 (10% vol), and further down to 38 mS/cm when glycerol (50% vol) is also included. However, this remains much higher than commonly reported for organic or ionic liquids electrolytes (∼1–10 mS/cm), and thus suitable for practical electrochemical cycling. This drop in ionic conductivity is accompanied by a substantial increase in viscosity, from 2.09 to 6.50 cP across the same series, which remains much lower than viscosity of pure glycerol (∼1500 cP) and is thus not expected to raise significant wettability issue. Accordingly, all subsequent data are reported for an electrolyte composed of 2 M KCl, 0.5 M Zn(OAc)_2_, 10% vol PEG‐200, and 50% vol glycerol (labelled K/Zn‐P_10_‐G_50_).

### 2.6 Spectroelectrochemical Cycling of GLAD‐PB Electrodes

The spectroelectrochemical features of the 1 cm^2^ (geometric surface area) GLAD‐PB electrodes were then investigated during galvanostatic cycling in a two‐electrode configuration, using the N_2_‐degassed optimized K/Zn‐P_10_‐G_50_ electrolyte and a zinc frame counter electrode. The GLAD‐PB electrodes were first in‐situ reduced to PW, and then subjected to successive charge–discharge cycles.

The evolution of the electrode transmittance was first analyzed by performing 10 galvanostatic charge–discharge (GCD) cycles at 1 mA/cm^2^, while recording the full transmittance spectra at the fully charged (colored) and discharged (bleached) states. The corresponding data for the 10th cycle are presented in Figures 3A–C and compared with those of a 2D‐PB‐10 electrode. The GLAD‐PB‐25 and GLAD‐PB‐50 electrodes can be reversibly cycled to their reduced PW state in the optimized electrolyte, presenting galvanostatic curves with well‐defined plateaus centered at 1.25 V versus Zn/Zn^2+^ and areal capacities of 30 and 45 mC/cm^2^, respectively. These values are consistent with those obtained above by cyclic voltammetry in KCl. During electrochemical cycling, the electrodes switch between a dark blue state (Figure 3B) and a transparent state (Figure 3C), leading to ΔT_633_ values of 80 and 55% for the GLAD‐PB‐25 and GLAD‐PB‐50 electrode, respectively. Notably, the transmittance of the reduced GLAD‐PB‐50 electrode remains ca. 20% lower than the reduced GLAD‐PB‐25, which is attributed to the moderate transparency of the thicker PW film. The high reversibility of the electrochromic response is confirmed by comparison of the PB spectra recorded before and after the 10th cycle (Figure 3B). It remains, however, to note that in the case of the GLAD‐PB‐100 electrode, the capacity that can be reversibly cycled at 1 mA/cm^2^ remains limited (<50 mC/cm^2^) compared to that previously achieved in the KCl electrolyte, and the film retains a blue coloration after the reduction step, resulting in only a minor increase in transmittance (ΔT_633_< 5%). Decreasing the current density is therefore necessary to reversibly cycle the entire PB coating, as demonstrated above by cyclic voltammetry. However, since a fast switching time remains essential for functional electrochromic devices, the GLAD‐PB‐100 electrode was not used for the further experiments.

**FIGURE 3:**
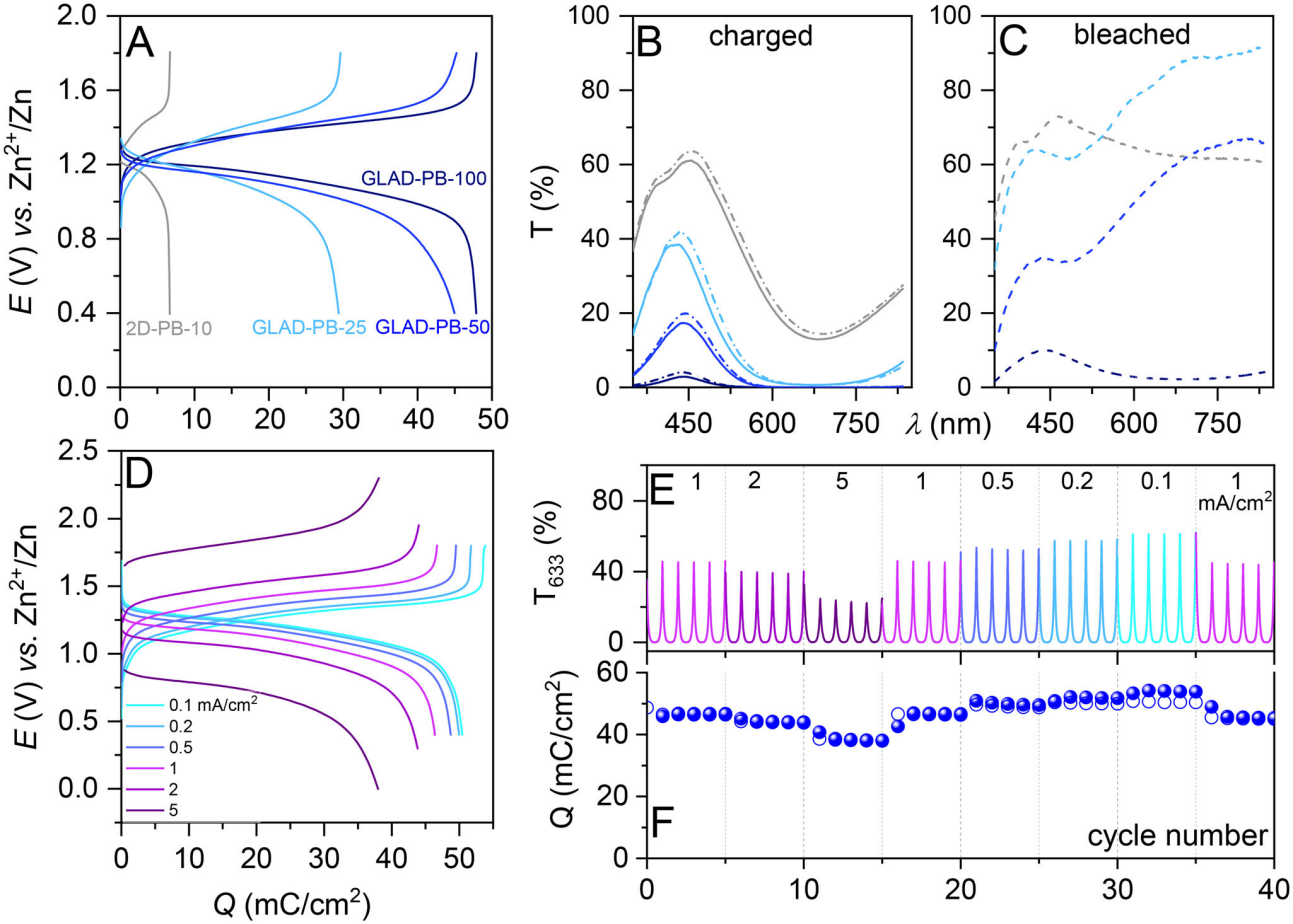
Spectroelectrochemical galvanostatic cycling of GLAD‐PB electrodes in the K/Zn‐P_10_‐G_50_ electrolyte. (A) GCD curves recorded at 1 mA/cm^2^ at (gray) 2D‐PB‐10, (light blue) GLAD‐PB‐25, (middle blue) GLAD‐PB‐50, and (dark blue) GLAD‐PB‐100 electrodes, along with transmittance spectra of the (B) charged (i.e., colored) and (C) discharged (i.e., bleached) electrodes, with the same color code as in A. In B, the solid lines correspond to the electrodes before cycling, while the dashed lines (in B and C) correspond to the 10th cycle. (D) GCD curves recorded at a GLAD‐PB‐50 electrode at different current densities. (E) Transmittance variation monitored at 633 nm upon cycling at the various current densities noted above the plots. (F) Charge (filled circles) and discharge (open circles) capacities obtained upon cycling.

In order to evaluate the rate performance of the GLAD‐PB‐50 electrode, galvanostatic cycling was initiated at 1 mA/cm^2^, after which the current density was progressively increased to 5 mA/cm^2^ and then progressively decreased to 0.1 mA/cm^2,^ with five cycles recorded at each current density. The corresponding GCD curves are shown in Figure 3D, while the transmittance simultaneously recorded at 633 nm is plotted in Figure 3E, and the corresponding charge/discharge capacities are gathered in Figure 3F. As the current density increases, both the capacity and the transmittance in the reduced state decrease, while the charge/discharge hysteresis increases significantly. These observations indicate incomplete film reduction at higher current density, along with a marked decrease in energy efficiency due to increased voltage hysteresis. Conversely, lowering the current density leads to slight increases in both the capacity and the transmittance of the reduced state, accompanied by reduced hysteresis. However, these improvements remain limited, and a current density of 1 mA/cm^2^ is a good compromise, enabling relatively fast cycling of the GLAD‐PB electrode (∼45 s per charge/discharge step for the GLAD‐PB‐50 electrode) while maintaining high capacity, limited hysteresis, and significant transmittance modulation.

Finally, the stability of the GLAD‐PB‐50 electrode was investigated in both its charged (colored/oxidized) and discharged (bleached/reduced) states, obtained after galvanostatic cycling (Figure S12). When cycling is stopped in the colored (charged) state and the electrode is thus monitored under open‐circuit voltage, both the coloration and electrode potential (>1.3 V vs. Zn^2^^+^/Zn) remain quite stable over 1 h. In contrast, when cycling is stopped in the bleached (discharged) state, the transmittance of the bleached device initially decreases before reaching a steady and highly stable value. Meanwhile, the open‐circuit voltage rapidly stabilizes at approximately 1.1 V versus Zn^2^^+^/Zn. This relaxation process is accompanied by an areal capacity loss of about 3% after a 1 h delay (and 7% after 15 h); however, both the initial transmittance and surface capacity were fully recovered upon subsequent cycling.

### 2.7 Smart Windows Assemblies

With the optimized electrolyte composition established, and the suitability of GLAD‐PB‐25 and GLAD‐PB‐50 electrodes confirmed as electrochromic cathodes, 6 and 25 cm^2^ smart windows were assembled using GLAD‐PB cathodes (with geometric surface areas of 3.2 or 17.6 cm^2^, respectively), zinc frame anodes, and Ar‐degassed K/Zn‐P_10_‐G_50_ electrolyte (see Experimental Section ‐ Supporting Information).

The resulting Zn//GLAD‐PB‐25 and Zn//GLAD‐PB‐50 smart windows were first subjected to chronoamperometric (CA) cycling between 0.4 and 1.8 V, with each potential applied for 50 s. The corresponding data are presented in Figures 4 and S13. The devices were reversibly switched between their colored (blue) and transparent states (see photographs in Figure 4B) over 50 consecutive cycles, maintaining a large and highly stable optical modulation at 633 nm (∼70% for the Zn//GLAD‐PB‐25 device) (Figure 4D and S13D). This excellent durability was accompanied by full opacity of the colored state across the 600–800 nm range (T < 0.5%) (Figure 4A). The switching times extracted from the transmittance evolution at 633 nm for 90% optical modulation reveal bleaching and coloration times of 3.9 and 3.1 s for the 6 cm^2^ Zn//GLAD‐PB‐25 device (Figure 4C). The Zn//GLAD‐PB‐50 (6 cm^2^) device shows similar trends. Because it incorporates more active material, the optical modulation at 633 nm is decreased to 55%, but it retains fast switching (6.5 s for bleaching and 2.4 s for coloration, see the blue data of Figure 4C), confirming the efficiency of the GLAD‐ITO substrate in rapidly switching the redox state of PB.

**FIGURE 4:**
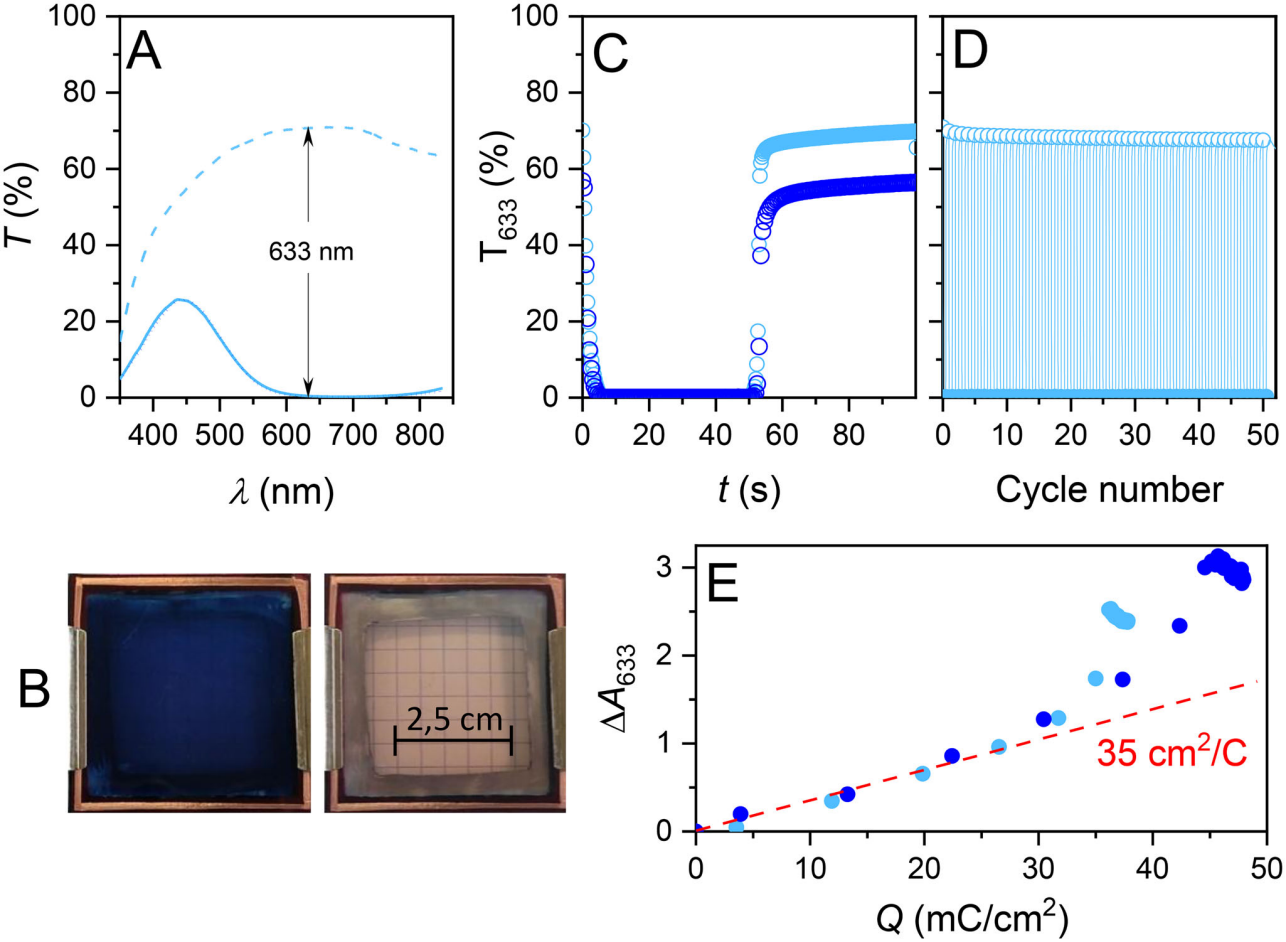
Chronoamperometric cycling of Zn//GLAD‐PB smart windows between 0.4 and 1.8 V. (A) UV‐visible transmission spectra of the Zn//GLAD‐PB‐25 device (6 cm^2^) during the first cycle, in its (plain light blue) initial, (dashed light blue) bleached, and (dotted blue line) re‐colored states. (B) Photographs of the 25 cm^2^ Zn//GLAD‐PB‐25 device in the (left) colored and (right) bleached states. (C, D) Time dependence of the transmittance at 633 nm for the 6 cm^2^ (light blue) Zn//GLAD‐PB‐25 and (darker blue) Zn//GLAD‐PB‐50 devices during (C) the first cycle and (D) over 50 continuous cycles. (E) Absorbance variation monitored at 633 nm as a function of the charge passed for the 6 cm^2^ (light blue dots) Zn//GLAD‐PB‐25 and (darker blue dots) Zn//GLAD‐PB‐50 devices.

For both 6 cm^2^ devices, the coloration efficiency, determined from the absorbance‐charge relationship (Figure 4E), follows Beer–Lambert behavior up to ≈25 mC/cm^2^, above which saturation occurs as the colored state approaches its limiting absorbance. From the linear regime, a coloration efficiency of 35 cm^2^/C is obtained. This value is lower than those usually reported in the literature for thin‐films of PB, whereas the switching times are of the same order of magnitude (Table 1). It should be noted, however, that literature values, ranging from 56.2 to 131.5 cm^2^/C [23, 29], rarely exhibit a perfectly linear absorbance‐charge relationship, so coloration efficiency is ultimately estimated over a very narrow charge range, resulting in somewhat optimistic values. Finally, upscaling the Zn//GLAD‐PB‐25 device (25 cm^2^) leads to bleaching/coloring processes occurring from the periphery to the center of GLAD‐PB‐25 electrode (Figure S13), while the overall magnitude of the transmittance variation remains unchanged compared to the 6 cm^2^ device. The inhomogeneous coloration/bleaching (i.e. iris effect) of the large Zn//GLAD‐PB‐25 device during chronoamperometric cycling is attributed both to inhomogeneous electric field lines resulting from the electrode geometry mismatch [48] and to the cycling mode itself, which favors high current intensities at the onset of the potential step and thus large Ohmic drops across ITO electrodes, owing to their limited electronic conductivity [49], together amplifying spatial inhomogeneity.

**TABLE 1:** Electrochromic and charge storage performance of Zn//PB electrochromic devices, obtained from chronoamperometric (CA) and chronopotentiometric (i.e., galvanostatic) cycling experiments, respectively. All values preceded by ∼ were estimated from published experimental data.

		CA cycling				CP cycling					refs.
Substrate	Electrolyte	Optical modulation @ 633 nm (%)	Coloration/Bleaching voltage (V)	Switching time t_C_/t_B_ (s)	Cycles	Discharge capacity (mAh.m^−2^)	Rate (A m^−2^); discharge time (s)	Average discharge voltage (V)	Retrieved energy density (mWh.m^−2^)	Cycles	
ITO	1 M KCl + 0.1 M ZnSO_4_ in PVA gel	67.2	1.6 / 0.8	3.6 / 2.5	1000	∼15	0.25^b^; ∼60	1.2	50	nr	[23]
FTO	0.9 M KCl + 0.1 M Zn(CH_3_COO)_2_	84.9	2 / 0	4.6 / 4.1	nr	78.9 62.7 35.7	0.07; 4058 0.7; 322 3.5; 37	1.24 ∼1.1	96 ∼39	nr 2000 nr	[24]
FTO	1 M hybrid KCl–ZnSO_4_	68.3 (700 nm)	1.6 / 0.8	4.7 / 7.5	1000	77.1 52.3	0.2; 1388 20; 9.4	∼1.2 ∼1.1	∼95 ∼57	nr 2000	[25]
FTO	1 M K(OTF) + 0.1 M Zn(OTF)_2_	55.3	1.8 / 0.4	2.8 / 2.6	1000	32.9 28.2	0.2; 592 2; 51	∼1.25 ∼1.2	∼41 ∼34	nr nr	[26]
FTO	0.2 M KCl	77.1 (630 nm)	2.0 / 0.3	4.1 / 3.2	nr	92.7 50.9	0.5; 667 4; 45.6	∼1.2 ∼1.1	∼111 ∼56	1000	[29]
PET‐ITO	1 M K(OTf) + 0.1 M Zn(OTF)_2_ QSS^a^	74.5 (700 nm)	1.8 / 0.2	26.7 / 19.9	10000	75.12 39.95	0.2; 1350 1.2; 120	∼1.2 ∼1.1	∼90 ∼44	nr 3000	[30]
GLAD‐ITO	2 M KCl + 0.5 M Zn(CH_3_COO)_2_ + PEG‐200 + Glycerol	55	1.8 / 0.4	2.4 / 6.5	50	133	10; 41	1.12	150	1060	This work

^a^QSS = quasi solid‐state electrolyte.

^b^Achieved with a chronoamperometric charge at 1.6 V for 10 s.

The Zn//GLAD‐PB‐50 smart window (6 cm^2^) was tested under galvanostatic charge/discharge cycling at 1 mA/cm^2^, following an initial PB cathode reduction step at 0.1 mA/cm^2^ to render the device transparent (the current here being normalized to the geometric surface area of the electrode). Three cycling sequences, that is, 50, 1000 and 10 cycles, were successively performed and the spectroscopic data recorded during the first and last sequences. As shown in Figure 5A, the GCD profiles are centered at 1.26 V versus Zn/Zn^2+^, with an initial discharge capacity of 48 mC/cm^2^ and an initial voltage hysteresis of 260 mV. During charging, transmittance decreases to full opacity in the 570–850 nm range (Figure 5B), and the optical modulation at 633 nm reaches 58%, remaining highly stable over 50 cycles (Figure 5E). The coloration efficiency of the device at 633 nm was estimated to be 63 cm^2^/C from the linear relationship between absorbance change and charge during the first charging step (Figure S14). This value is higher than that obtained from chronoamperometric cycling (see above), which may indicate a more homogeneous coloration process when its rate is controlled through the application of a constant current density. The Coulombic efficiency increases slightly during early cycling and then stabilizes above 99.9% (Figure 5D). After 1060 cycles, the charged‐state transmittance remains essentially unchanged, staying opaque over a broad spectral range (Figure 5A), while the discharged capacity decreases to 75% (Figure 5C), leading to lower transparency in the discharged state (green line in Figure 5B) and an optical modulation retention of 73% (Figure 5E). Such an optical modulation retention is in line with what is usually reported for Zn//PB smart windows assemblies (78% after 100 CA cycles in ref [25]; 78.7% after 2000 GCD cycles at 0.7 A/m^2^ in ref [24]).

**FIGURE 5:**
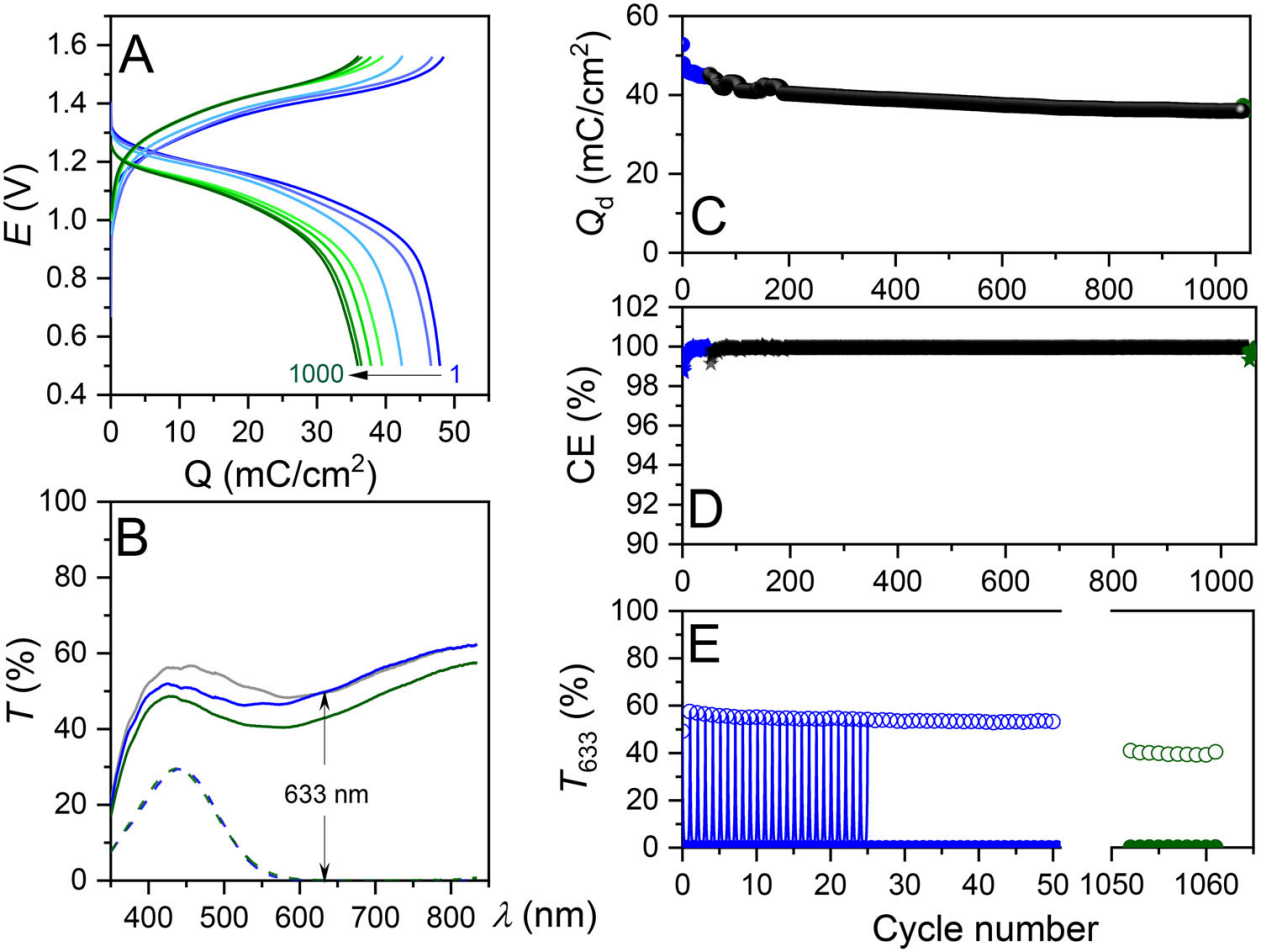
Galvanostatic cycling of the Zn//GLAD‐PB‐50 smart window between cut‐off voltages fixed at 0.5 and 1.56 V, at 1 mA/cm^2^, over three successive sequences comprising 50 cycles, 1000 cycles, and 10 cycles. (A) GCD curves for (from blue to dark green) cycles 1, 25, 100, 250, 500, 750, and 1000. (B) UV‐visible transmission spectra of (gray) the pristine discharged device, (blue) the first charge–discharge cycle, and (green) the 1051th cycle, with charged and discharged states shown as dashed and solid lines, respectively. (C) Discharge capacity, (D) Coulombic efficiency, and (E) transmittance monitored at 633 nm upon cycling, with data from the sets of 50, 1000 and 10 cycles marked in blue, black, and green, respectively.

The overall electrochromic and charge storage performance of the Zn//GLAD‐PB‐50 smart window is summarized in Table 1. The device delivers an initial energy density of 150 mWh/m^2^ at a high current density of 10 A/m^2^, corresponding to a full charge–discharge (coloration‐bleaching) cycle in less than 100 s with 78% round‐trip energy efficiency. These charge storage metrics outperform state‐of‐the‐art devices, with are limited by low PB loading and poor rate capability. The superior performance of the Zn//GLAD‐PB‐50 device can be attributed to the nanostructured GLAD‐ITO electrode, which enables (i) stable electrodeposition of high PB loadings owing to its large 3D‐anchoring surface area, and (ii) minimized ohmic losses across the PB layer (the latter having a well‐known limited electronic conductivity [50]), thereby achieving high areal capacity.

To further enhance transmittance modulation and scalability, a 6 cm^2^ dual‐tinting smart window was assembled, exploiting the reversible Zn electrodeposition on a transparent negative ITO anode instead of on a nontransparent zinc frame. For this purpose, an ITO//GLAD‐PB‐50 device was assembled under controlled atmosphere, incorporating face‐to‐face a GLAD‐PB‐50 cathode pre‐reduced to PW and a commercial ITO electrode (see Experimental Section ‐ Supporting Information). Upon anodic charging (1 mA/cm^2^), the transmittance dropped from an initial value of ca. 40% to below 5% across the visible range (Figure S15), demonstrating improved optical contrast compared with the single‐tinting Zn//GLAD‐PB‐50 device described above. Notably, the transmittance at 430 nm is significantly lower than that of the Zn//GLAD‐PB‐50 device, as a consequence of reversible Zn electrodeposition at the ITO counter electrode (compare the black and blue curves in Figure S15B). To further highlight the optical contribution of reversible zinc electrodeposition, the coloration efficiencies of single‐ and dual‐tinting devices at 633 and 430 nm are compared in Figure S14. The dual tinting device exhibits an increase in coloration efficiency of 27% at 633 nm and 350% at 430 nm. During galvanostatic cycling, however, both transmittance modulation and the areal capacity degraded, owing to an inadequate Coulombic efficiency of ca. 98%, causing progressive PB accumulation at the cathode. It is worth noting that reversible Zn electrodeposition on transparent conductive oxide electrodes from aqueous electrolytes remains challenging [45, 51, 52].

To address these issues, we developed a 6 cm^2^ three‐electrode smart window incorporating a sacrificial zinc frame. This frame, sandwiched between a face‐to‐face positive GLAD‐PB‐50 electrode and a negative planar transparent ITO electrode, enables in situ prereduction of the GLAD‐PB‐50 cathode, facilitates clear differentiation between optical states, and allows full recovery of transparency through an appropriate rejuvenation mode as detailed below. After in‐situ galvanostatic reduction of the GLAD‐PB‐50 cathode (i.e., after applying a 43.6 mC/cm^2^ discharge against the Zn frame at ‐0.1 mA/cm^2^), the device appears nicely transparent, despite a transmittance below 40% in the visible range (Figure 6B and photographs in Figure 6C). The reduced GLAD‐PB electrode was next cycled against the planar ITO counter‐electrode and subjected to successive charge–discharge cycles at 1 mA/cm^2^. Upon charging, the transmittance of the smart window decreases below 5% across the visible range, and below 0.1% at 633 nm, confirming its high opacity and the benefit of dual‐tinting (see photograph in Figure 6C, and Video S1 of the first cycle). As for the ITO//GLAD‐PB‐50 device, the Coulombic efficiency increases to ca. 98% upon cycling, while the capacity and transmittance of the discharged state continuously decrease, associated with the accumulation of a residual blue coloration at the cathode. Interestingly, incorporating a slow in‐situ galvanostatic discharge (at ‐0.1 mA/cm^2^) of the GLAD‐PB‐50 cathode against the Zn frame at the end of a series of 20 charge/discharge cycles allows recovery of both the initial transmittance as well as capacity of the device, enabling further successful cycling. This in‐situ rejuvenation strategy could be repeated to achieve up to 100 cycles without significant loss of performance. This clearly provides lab‐scale proof‐of‐concept that incorporating in‐situ rejuvenation modes improves the device's cyclability, and that optimizing the overall cycling protocol is a valuable strategy for achieving long‐term cycling stability. Implementing such a protocol would, however, require automating the switching of electrode connections, adding complexity and cost to the device's control electronics, a nontrivial constraint for the practical deployment of smart windows.

**FIGURE 6:**
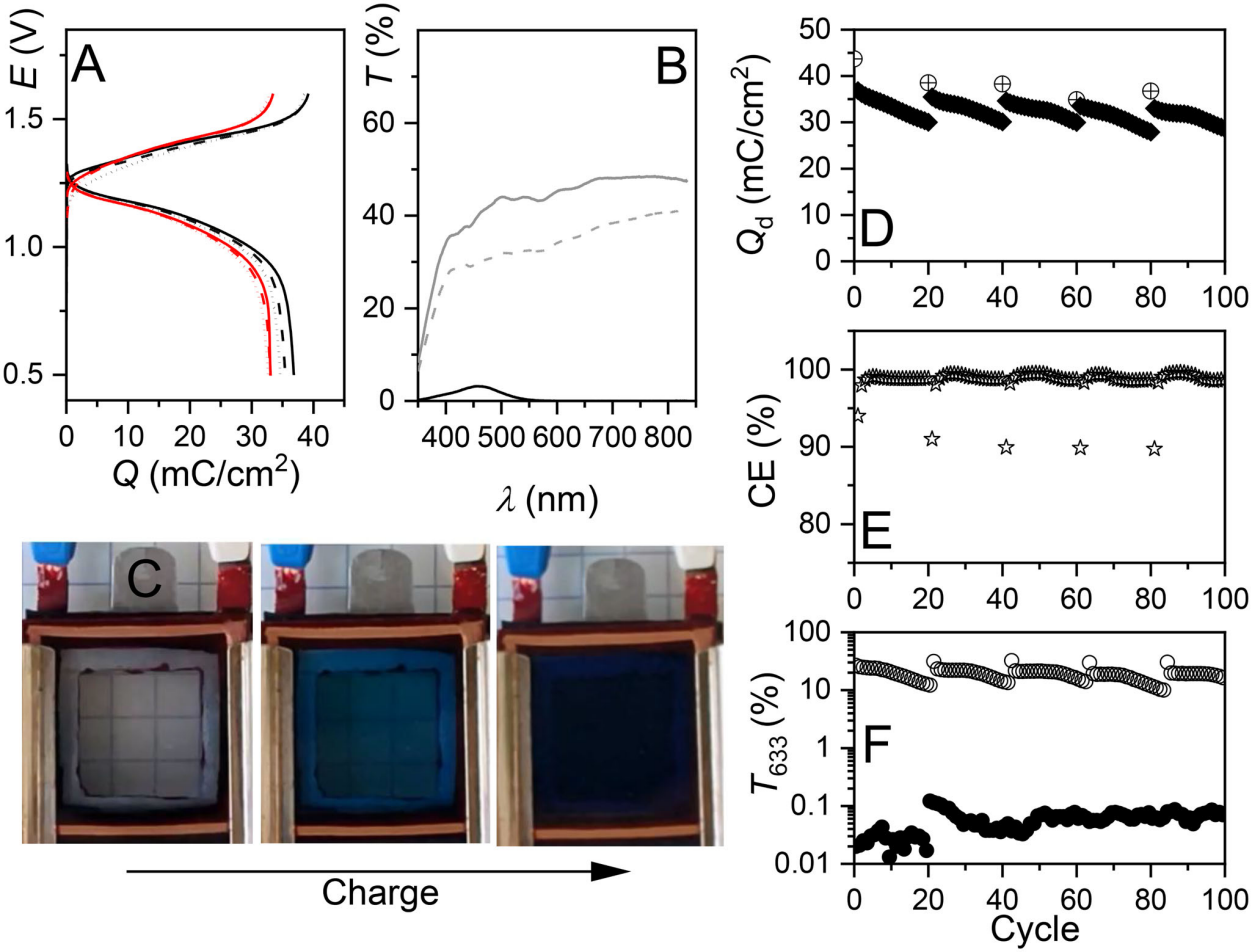
Galvanostatic cycling of the dual‐tinting ITO//Zn//GLAD‐PB‐50 smart windows (6 cm^2^) at 1 mA/cm^2^, between cut‐off voltages fixed at 0.5 V and 1.56 V, over 5 successive sequences, each incorporating an in‐situ discharge of the GLAD‐PB‐50 electrode against Zn at 0.1 mA/cm^2^, followed by 20 successive charge/discharge cycles against ITO at 1 mA/cm^2^. (A) GCD curves for the (black) first and (red) 10th cycle of the (plain line) first, (dashed line) second, and (dotted lines) third cycling sequences. (B) UV‐visible transmission spectra of (solid gray) the pristine discharged device, and (solid black) the first charged and (dotted gray line) discharged device. (C) Photographs of the dual‐tinting device during charging. (D) Discharge capacity, (E) Coulombic efficiency, and (F) transmittance monitored at 633 nm upon cycling.

Finally, the three‐electrode ITO//Zn//GLAD‐PB‐50 device was successfully scaled up to 25 cm^2^. As shown in Video S2, cycling the GLAD‐PB‐50 electrode against the ITO counter electrode (following an initial in situ reduction versus the Zn frame) results in homogeneous coloration and bleaching, with a complete charge–discharge cycle achieved in less than 2 min. Because the current density was kept constant at 1 mA/cm^2^ (normalized to the geometric surface area) to maintain fast cycling conditions, upscaling inherently leads to an increase in the absolute current. However, coloration/bleaching remains homogeneous, indicative of minor Ohmic drop and voltage gradients across the ITO electrodes [49]. Upon galvanostatically cycling the GLAD‐PB‐50 cathode against the Zn frame in the same device, an iris effect remains, as shown in Video S3, although it is less marked than upon chronoamperometric cycling (Figure S13). These control experiments underscore the advantages of using two electrodes of identical size, arranged face‐to‐face in parallel to promote a more uniform distribution of electric field lines within the device, and of relying on a galvanostatic cycling mode, in which the current amplitude is controlled and limited. Upon further upscaling the device, decreasing the current density should be a valuable strategy to mitigate uneven coloration/decoloration, if any, albeit at the expense of the switching time.

## 3 Conclusion

In conclusion, this work demonstrates the advantages of using high surface area nanostructured transparent electrodes for the conformal and homogeneous electrodeposition of an electrochromic material such as Prussian Blue, thereby overcoming the mechanical stability limitations generally encountered with conventional planar transparent conductive oxide electrodes. The 1 µm‐thick GLAD‐ITO electrodes loaded with Prussian Blue present a good cycling stability and rate performance.

The resulting electrodes were successfully integrated into energy‐storage smart windows coupled with reversible zinc electrodeposition, either at a Zn frame or at a planar transparent ITO counter electrode, yielding systems capable of fast and reversible coloration/bleaching under both chronoamperometric and galvanostatic (i.e., chronopotentiometric) conditions. These devices further exhibit charge storage capacities that significantly exceed the current state of the art for single‐tinting electrochromic systems.

Finally, we demonstrate a fully operational dual‐tinting configuration based on reversible Zn electrodeposition at an ITO counter electrode, representing a key step toward improved opacity in the charged state and a more homogeneous distribution of electric field lines within the device. In addition, a three‐electrode configuration incorporating a sacrificial zinc frame is proposed to enable in situ regeneration of the cathode, thereby further enhancing the versatility and durability of the system.

## Author Contributions

**Renald Sangarane**: methodology, investigation, formal analysis, writing–original draft, visualization. **Hamid Palamadathil Kannattil**: investigation. **Kenneth D. Harris**: funding acquisition, resources, writing ‐ review and editing. **Benoît Limoges**: conceptualization, funding acquisition, writing ‐ review and editing. **Véronique Balland**: conceptualization, methodology, investigation, supervision, project administration, funding acquisition, writing ‐ review and editing, writing ‐ original draft.

## Conflicts of Interest

The authors declare no conflicts of interest.

## Supporting information




**Supporting File 1**: smtd71123‐sup‐0001‐SuppMat.docx.

**Supporting File 2**: smtd71123‐sup‐0002‐VideoS1.mp4.

**Supporting File 3**: smtd71123‐sup‐0003‐VideoS2.mov.

**Supporting File 4**: smtd71123‐sup‐0004‐VideoS3.mov.

## Data Availability

The data that support the findings of this study are available from the corresponding author upon reasonable request.
