# Supplementary material for: 3D‐Nanostructured ITO Enables High‐Loading Prussian Blue Cathodes for Zn‐Based Electrochromic Smart Windows
Source: Small Methods. 2026 Oct 15;10(21):e71123. doi: 10.1002/smtd.71123 (PMC13745970; doi:10.1002/smtd.71123)
Supplement: Supplementary file 1 [file SMTD-10-e71123-s002.docx]

Supplementary Information to

3D-Nanostructured ITO Enables High-Loading Prussian Blue Cathodes for Zn-Based Electrochromic Smart Windows

Renald Sangarane,^1^ Hamid Palamadathil Kannattil,^1^ Kenneth D. Harris,^2^ Benoît Limoges,^1^ Véronique Balland^*,1^

1. Université Paris Cité, CNRS, ITODYS, Paris F-75013, France

2. National Research Council Canada – Quantum and Nanotechnologies Research Centre, Edmonton, AB, Canada and Department of Mechanical Engineering, University of Alberta, Edmonton, AB, Canada

Corresponding author : veronique.balland@u-pariscite.fr

I. **Experimental section**

**Chemicals.** All chemicals were purchased from commercial suppliers and used without further purification. ZnCl₂, Zn(OOCH₃)₂·2H₂O (>99.5%), KCl (>99.5%), K₃[Fe^III^(CN)₆] (>99%), FeCl₃·6H₂O (>99.5%), polyethylene glycol (PEG-200), and ethanol (>99.5%) were obtained from Sigma-Aldrich; glycerol (>99%) was obtained from Thermo Fisher Scientific; dichloromethane (100%), acetone (100%), and HCl (37%) were obtained from VWR Chemicals. Zinc metal foils (>99.5%, 0.25 mm thick) were purchased from Goodfellow. Copper adhesive tapes (3M 1181) were purchased from RS. Commercial 2D-ITO electrodes (12 Ω/□) were obtained from SOLEMS. All aqueous electrolytes were prepared from Milli-Q ultra-pure water (18.2 MΩ·cm).

**GLAD electrodes.** The mesoporous ITO film was deposited by glancing angle deposition (GLAD) on 0.9 × 2.5 cm^2^ (alternatively 2.5 × 2.5 cm^2^ and 5 × 5 cm^2^) commercially-sourced glass/ITO substrates (Delta Technologies). The substrates were cleaned by hand in soapy water, successively sonicated in water and isopropanol, then blown dry in an N_2_ stream. The substrates were then mounted on an aluminum deposition plate using Kapton tape to mask ~2.5 mm wide areas at the top of the 0.9 × 2.5 cm^2^ substrates, and around each edge of the square substrates. Accordingly, ITO film was deposited within the ca. 2 × 2 cm^2^ (alternatively ca. 4.5 × 4.5 cm^2^) interior, while the perimeter remained uncoated. The deposition plate was loaded into a GLAD-equipped and feedback-controlled electron-beam evaporation system (Kurt J. Lesker), and ITO pellets (Materion, 91:9 In_2_O_3_/SnO_2_, 99.99%) were placed in a graphite crucible liner and inserted into the e-beam source. Prior to deposition, the vacuum chamber was evacuated to < 5 x 10^-5^ Pa, and during deposition, a 7.5 kV electron accelerating voltage was applied while the e-beam current (~40-60 mA) and position were continuously adjusted to maintain a ~0.4 nm/s deposition rate as measured by quartz crystal microbalance. Post-like structures were formed by positioning the deposition plate at a fixed 85° angle with respect to the direction of ITO vapor incidence, and rotating the plate at a rate of 1 revolution per 10 nm of deposited film. Following deposition, the GLAD-ITO electrodes were subjected to a two-stage anneal process to improve both transparency and electrical conductivity. First, the electrodes were annealed in an air environment at 500°C for 1.5 hrs, followed by annealing in forming gas (5% H_2_/Ar) at 300°C for 1 hr.

**Electrode preparation.** Soon before use, the GLAD-ITO electrodes were cleaned by successive immersion in chloroform, acetone, and ethanol at 50°C for 30 min each. The commercial 2D-ITO electrodes were cleaned by sonication in successive baths of dichloromethane, acetone, and ethanol (5 min each). To improve electrical connectivity, a conductive copper tape was applied to the outer edges of all transparent electrodes, and the tape was subsequently covered with an insulating layer to ensure no Cu ions contaminate the electrolyte. With 2.5x2.5 cm^2^ substrates (i.e., 6.25 cm^2^ devices), the resulting delimited geometric surface area was 1.8x1.8 cm^2^ (i.e., 3.2 cm^2^). With 5x5 cm^2^ electrodes (i.e., 25 cm^2^ devices), the resulting delimited geometric surface area was 4.2x4.2 cm^2^ (i.e., 17.6 cm^2^).

**PB electrodeposition.** Modified electrodes were prepared following the method reported by Itaya^1^ *via* galvanostatic electrodeposition onto 2D-ITO and GLAD-ITO substrates, in a conventional three-electrode cell using a platinum wire counter electrode and a saturated Ag/AgCl (KCl) reference electrode. The deposition electrolyte was made of 0.01 M K₃[Fe(CN)₆], 0.01 M FeCl₃·6H₂O, and 0.05 M KCl. Prussian Blue films were electrodeposited galvanostatically at a current density of –50 µA cm⁻² for durations fixed to achieve the appropriate electrodeposition charge. The resulting electrodes were thoroughly rinsed with ultrapure water and heated overnight at 60°C.

**Dynamic window assemblies.** *Single-tinting Zn//GLAD-PB assemblies* were assembled by combining a GLAD-PB electrode with a butyl rubber frame as first separator, a Zn metal frame as the counter electrode, a second butyl rubber frame as second separator, and a microscope slide as back glass window. *Dual-tinting Zn//GLAD-PB assemblies* were assembled in a glove box by combining a reduced GLAD-PB electrode, one frame of butyl rubber, and a 2D-ITO counter electrode. *ITO//Zn//GLAD-PB assembly with sacrificial zinc anode* was assembled by combining a GLAD-PB electrode, one frame of butyl rubber, a zinc frame, a second butyl rubber frame and a 2D-ITO counter electrode. All windows were sealed with clips, and filled with deaerated K/Zn-P_10_-G_50_ electrolyte solution.

**Morphology characterization and spectroscopic techniques. SEM:** Surface morphology (Fig. S3) and EDX Elemental analyses were performed using an Oxford -Ultim Max 170 mm^2^ detector-integrated into a GeminiSEM 360 scanning electron microscope. Data were collected under high vacuum at an accelerating voltage of 10 kV. Alternatively, top-view and cross-sectional SEM imaging (Fig. 1 & Fig. S8) was performed using an ultra-high-resolution Hitachi S-5500 SEM at an accelerating voltage of 1.5 kV. **XRD:** Crystallographic analysis was carried out on a Bruker D8 Advance diffractometer in Bragg–Brentano geometry, equipped with a copper microfocus source (Cu Kα radiation, λ = 1.54 Å) and a Vantec 500 surface detector. XRD patterns were recorded at room temperature over a 2θ range of 5–100°, using a step size of 0.05° and a counting time of 300 s per step. Crystallographic analysis was also performed using an Empyrean multifunction diffractometer (Malvern Panalytical), equipped with a copper source, a multichannel detector, and a 5-axis sample stage for optimized sample alignment. **XRF:** X-ray fluorescence measurements were performed using an energy-dispersive Epsilon 3XL spectrometer (Malvern Panalytical) equipped with a silver tube and helium purging to improve the signal from light elements. **Raman Spectroscopy:** Raman spectra were recorded using a LabRAM HR800 spectrometer (Horiba Jobin-Yvon) equipped with a 633 nm He–Ne excitation laser. A 300 lines mm⁻¹ holographic grating was used to achieve optimal spectral resolution and sensitivity. Measurements were carried out using a ×100 objective lens (NA = 0.9). The laser power was set to 0.76 mW (10%), with an acquisition time of 20 s and 3 accumulations, in order to optimize signal quality while avoiding sample damage. The scattered light was collected and directed to a CCD detector for spectral acquisition.

**Electrolyte characterization.** All measurements were performed at 22°C. Ionic conductivity was measured with an Accumet XL200 benchtop meter (Fisher Scientific) calibrated using standard KCl solutions. Dynamic viscosity was measured using a Brookfield LVDV2T rotational viscometer equipped with a CPA-40Z cone. The viscosity values were acquired at rotational speed values ranging from 45 to 140 RPM, and repeated several times to calculate the mean value and the standard deviation.

**Electrochemistry.** All electrochemical experiments were carried out using a VSP BioLogic potentiostat controlled by EC-Lab 11.36 software. Electrochemical analysis of the modified electrodes was performed in a conventional three-electrode cell using a platinum wire counter electrode and a saturated Ag/AgCl (KCl) reference electrode, containing a degassed 3 M KCl electrolyte, or an electrolyte of adjusted composition for electrolyte optimization. In all experiments, the current density refers to the geometric surface area of the electrode considered.

**UV-visible absorption spectroscopy**. The visible-NIR transmittance spectra were recorded over 360-825 nm using a UV-visible optical fiber connected to an Ocean Optics TORUS Concave Grating Spectrometer controlled by SpectraSuite software. The integration time was set at 3 ms, with an average of 100 scans. Light was provided by a DH-2000-BAL halogen lamp (Ocean Insight).

**Spectroelectrochemistry.** All electrochemical experiments were carried out using a VSP BioLogic potentiostat controlled by EC-Lab 11.36 software. Spectroelectrochemical analysis of the modified PB electrodes was first achieved on small electrodes (0.9 x 2.5 cm^2^) in a degassed quartz cuvette incorporating a zinc counter electrode, with the blank spectrum being recorded on the cell filled with the electrolyte. For smart windows assemblies, blank spectra were recorded in air.

The optical contrast retention (OCR in %) of the device is defined as

$$OCR(\lambda)=\frac{\log\left( \frac{T_{discharged,i}}{T_{charged,i}} \right)}{\log\left( \frac{T_{initial}}{T_{charged,1}} \right)}\times100$$

where $T_{discharged,i}$ and $T_{charged,i}$ are the transmittance of the fully discharged and charged device for cycle *i*, respectively, $T_{initial}$ is the transmittance of the device in its initial discharged state, and $T_{charged,1}$ is the transmittance of the device after the first charge. All transmittance values are monitored at an identical wavelength *λ*.

**Charge storage performances.** The Coulombic efficiency (*CE* in %) for each galvanostatic cycle is defined as

$$CE=100\times\frac{Q_{discharge}}{Q_{charge}}$$

where $Q_{discharge}$ (resp. $Q_{charge})$ is the amount of charge (in mC/cm^2^) passed upon discharge (resp. charge).

The energy density (in mWh/m^2^) was calculated from integration of the galvanostatic curve, corresponding thus to the following equation:

$$E=i\times\int Vdt$$

where *i* is the (fixed) intensity (in mA/m^2^) and *V* the discharge voltage (in V).

The energy efficiency (*EE* in %) for each galvanostatic cycle is defined as

$$EE=100\times\frac{E_{discharge}}{E_{charge}}$$

where $E_{discharge}$ (resp. $E_{charge})$ is the discharge (resp. charge) energy density (in mWh/m^2^) calculated from integration of the discharge (resp. charge) galvanostatic curve.

II. **Supplementary Tables**

**Table S1.** Chemical composition of the bare and modified electrodes assessed by EDX analysis. Each data is the average of 2-4 measurements at different spots. Data are also given for a 2D-PB-10 electrodes after the stabilization process (i.e., 20 CVs performed in a KCl electrolyte of pH 3).

| ITO Substrate | 2D-ITO | 2D-ITO | 2D-ITO | GLAD-ITO | GLAD-ITO | GLAD-ITO |
| --- | --- | --- | --- | --- | --- | --- |
| *Q*_ED_ (mC/cm^2^) | 0 | 10 | 10  after stabilization | 0 | 25 | 50 |
| Element | average atomic (%) | average atomic (%) | average atomic (%) | average atomic (%) | average atomic (%) | average atomic (%) |
| C | 2.34 | 23.78 | 28.94 | 2.48 | 27.93 | 38.04 |
| N | 15.1 | 24.01 | 25.94 | 17.88 | 28.91 | 33.33 |
| O | 48.96 | 19.87 | 16.03 | 45.81 | 16.61 | 6.5 |
| K | 0.28 | 0.46 | 1.05 | 1.23 | 0.49 | 0.32 |
| Fe | 2.38 | 18.14 | 17.88 | 2.94 | 16.27 | 19.05 |
| In | 28.80 | 12.71 | 9.40 | 28.05 | 9.28 | 2.72 |
| Sn | 2.15 | 1.02 | 0.75 | 1.61 | 0.51 | 0.04 |
| Total | 100 | 100 | 100 | 100 | 100 | 100 |
| **C/N** |  | **0.99** | **1.12** |  | **0.97** | **1.14** |
| **K/Fe** |  | **0.025** | **0.058** |  | **0.030** | **0.017** |

III. **Supplementary Figures**

**Figure S1.** (A) Cyclic voltammograms of 1 μm-thick GLAD-ITO in 1 M KCl recorded (from yellow to violet) at 0.03, 0.06, 0.09, 0.12 and 0.15 V/s. (B) Evolution of the current density (normalized to the geometric electrode area) with the scan rate for GLAD-ITO (blue) and 2D-ITO (black). Dashed red lines correspond to the linear regressions of the experimental data, with slopes of 256 and 16 μF/cm^2^ at GLAD-ITO and 2D-ITO, respectively.

**Figure S2.** Photographs of the 1 μm-thick GLAD-ITO electrodes, prior to and after PB electrodeposition.

**Figure S3.** (Top) SEM images and (bottom) EDX spectra of the (A, D) 2D-PB-10 electrode, (B, E) GLAD-PB-25 electrode, and (C, F) GLAD-PB-50 electrode. The chemical composition of the film is detailed in Table S1.

**Figure S4.** Galvanostatic charging curve monitored during electrodeposition of PB at (left) 2D-ITO and (right) GLAD-ITO electrodes. At 2D-ITO electrodes, experiments were performed with different cathodic intensities, from -50 to -1.5 μA/cm^2^. At GLAD-ITO electrodes, data are shown for a series of 18 electrodes, with *Q*_ED_ ranging from 25 to 100 mC/cm^2^, demonstrating the high reproducibility of electrodeposition.

**Figure S5.** (A) Mass of Fe on the modified electrodes as measured by XRF plotted as a function of the charge passed during electrodeposition at (grey dots) 2D-ITO and (empty dots) GLAD-ITO electrodes, along with (red lines) the associated linear regressions (slope = 1.13 μg/cm^2^, intercept fixed at 5 μg/cm^2^ according to measurements on bare GLAD-ITO electrodes, R^2^ = 0.990). Empty stars correspond to modified electrodes after preconditioning (see text). (B) Thickness of the dense PB film as a function of the charge passed during electrodeposition on the 2D-ITO electrode (grey dots) and outside the GLAD-ITO film thickness (empty dots). Thicknesses were determined from the cross-section SEM images (see Figures 1B, 1E and 1F). The red lines correspond to the linear regressions with slopes of 0.017 and 0.016 μm∙cm^2^/mC on (plain line) 2D-ITO and (dashed line) GLAD-ITO, respectively.

**Figure S6**. (A) X-Ray diffractograms of the (black) modified 2D-ITO, (grey) unmodified GLAD-ITO, and (blue) modified GLAD-ITO electrodes, along with (red) PDF Card 01-073-0689 for cubic Fm-3m Prussian Blue (formula $\mathrm{Fe}_{4}^{\mathrm{III}}\left[ \mathrm{Fe}^{\mathrm{II}}{(CN)}_{6} \right]_{3}, xH_{2}O$) and (light grey) PDF Card 98-005-0846 for cfc ITO. (B) Low-frequency and (C) high-frequency Raman spectra of the modified 2D-ITO and GLAD-ITO electrodes.

**Figure S7.** Electrode preconditioning in a 3 M KCl aqueous electrolyte of pH 3, by recording 20 successive cyclic voltammograms (from blue to red) at 20 mV/s, at (A) 2D-ITO-PB-10, (B) GLAD-PB-25, (C) GLAD-PB-50, and (D) GLAD-PB-100 electrodes. (E) and (F) show the capacity of all four electrodes extracted from (empty dots) reduction and (plain dots) oxidation peak integrations.

**Figure S8.** (A, B) Side-view and (C, D) top-view SEM images of the GLAD-PB-25 electrodes, (A, C) before and (B, D) after preconditioning in a 3 M KCl electrolyte of pH 3.

**Figure S9**. Cyclic voltammograms recorded at the modified electrodes for scan rates *v* ranging between (from red to blue) 20 and 0.05 mV/s in a 3 M KCl aqueous electrolyte of pH 3 (the current densities were normalized to *v*). It can be noted that at extremely low scan rates, a cathodic current intensity remains at the reverse potential of -0.2 V, giving rise to an increased charge passed during the cathodic scan and a low coulombic efficiency of the CV (this is especially true for the GLAD-PB-100 electrode). This may be due to some O_2_ reduction,^2^ the presence of the latter resulting either from minor leaks in the setup or from oxygen release by the modified electrode itself, as it was conditioned in air.

**Figure S10**. (A) (Grey) experimental and (red) simulated CV of the 2D-PB-10 electrode. The experimental CV corresponds to a scan rate of 0.2 mV/s. (B) (Blue) experimental and (dashed red line) reconstructed CV of the GLAD-PB-50 electrode at 0.1 mV/s, along with experimental CVs recorded at 0.2 mV/s at the (grey) 2D-PB-10 and (light blue) GLAD-PB-25 electrode. (C) (Black) experimental and (dashed lines) reconstructed CVs of the GLAD-PB-100 electrode at 0.1 mV/s, considering either (dashed red line) GLAD-PB-25 and 2D-PB-10 contributions or (dashed blue line) only GLAD-PB-50 contribution (*note that the reconstructed CVs were adjusted to the baseline of the GLAD-PB-100 CV*).

**Comment :** The current density $j$ (in A/cm^2^) recorded at the electrode is related to the time derivative of the mole fractionof the oxidized PB state $\theta$ according to :

$$j=F\times\Gamma_{0}\times\frac{d\theta}{dt}$$

Where $\Gamma_{0}$ is the surface coverage of redox-active sites (mol/cm^2^), such that $F\times\Gamma_{0}= Q$ is the surface capacity (C/cm^2^) of the electrode.

Thus, the CV of the ITO-PB-10 electrode under thermodynamic equilibrium conditions (with current density normalized to the scan rate, in C/(V.cm^2^)) can be simulated by differentiating the Nernst equation 3 to calculate $\frac{dE}{d\theta}$ (see main text), which in turn allows the calculation of :

$$\frac{j}{v}=Q\times\frac{d\theta}{dE}$$

The simulation obtained using $E_{PB/PW}^{0}$= 0.274 V *vs*. Ag/AgCl, $a_{K_{aq}^{+}}=1.94$(considering a 3 mol/L concentration equivalent to a 3.5 mol/kg)^3^, *g* = -3, and *Q* = 6.6 10^-3^ C/cm^2^, closely matches the experimental CV, with a fwhm value of 15 mV for the faradaic peaks. However, the Frumkin isotherm fails to capture the observed asymmetry in fwhm values between the cathodic and anodic faradaic peaks, as well as the associated potential hysteresis.

It is worth noting that the charge used for the simulation corresponds to 60% of the total charge passed upon cycling the ITO-PB-10 electrode (*i.e.*, a total of 11 mC/cm^2^). This discrepancy can be explained by the large background current present in the experimental CV, which contributes to the total integrated charge.

**Figure S11**. Galvanostatic charge-discharge curves for 2D-PB-ITO electrodes soaked in aqueous electrolytes containing (blue) 3 M KCl, (red) 1 M Zn(CH_3_COO)_2_, and (black) 2 M KCl + 0.5 M Zn(CH_3_COO)_2_, and cycled at (A) 1 and (B) 0.1 mA/cm^2^.

Note: the slight downshift of the curves in the mixed electrolyte compared to the pure KCl electrolyte is consistent with its lower K^+^ concentration (according to the Nernst law) and in line with a charge storage mechanism associated with reversible K^+^ insertion, despite the presence of Zn^2+^.

**Figure S12**. Evolution of the transmittance (monitored at 633 nm) and the open circuit voltage (*vs*. Zn^2+^/Zn counter electrode) of (left) a discharged/bleached and (right) charged/colored GLAD-PB-50 electrode. After a series of 10 galvanostatic cycles were performed at 1 mA/cm^2^ in the spectroelectrochemical cell, the electrode was left to spontaneously evolve over 1 h under open-circuit voltage.

**Figure S13.** Chronoamperometric cycling of the 25 cm^2^ Zn//GLAD-PB smart windows between 0.4 V and 1.8 V. (A) UV-visible transmission spectra recorded at the center of the device in its (plain violet) initial state, (dashed violet line) bleached state, and (dotted dark violet line) re-colored state. (B) Photographs taken during the bleaching/coloring cycle. (C, D) Time-dependence of the transmittance at 633 nm during the first cycle and over 50 continuous cycles. From C, the switching times are estimated to be 12 s at the center of the electrode, and 4.5-6.5 s at the corners of the electrode.

**Figure S14.** Absorbance monitored at 633 and 430 nm during first charge (*i.e.* coloration) of the (blue) single-tinting Zn//GLAD-PB-50 device and (black) dual-tinting ITO//GLAD-PB-50 device.

**Figure S15.** Galvanostatic cycling of the dual-tinting ITO//GLAD-PB-50 smart windows (6 cm^2^) at 1 mA/cm^2^ between cut-off voltages fixed at 0.5 V and 1.56 V, after a first *in-situ* discharge of the GLAD-PB-50 electrode against Zn at 0.1 mA/cm^2^. (A) Charge-discharge curves for cycle 1 (black) to 50 (blue), every 5^th^ cycle being shown. (B) UV-visible transmission spectra of the ITO//GLAD-PB-50 device in its (solid grey) pristine discharged state, (black) first charged state, and (dashed grey line) first discharged state, along with (solid blue line) charged and (dashed blue line) discharged states of the Zn//GLAD-PB-50 device. (C) Discharge capacity, (D) Coulombic efficiency, and (E) transmittance monitored at 633 nm upon cycling.

IV. **References**

(1)	Itaya, K.; Ataka, T.; Toshima, S. Spectroelectrochemistry and Electrochemical Preparation Method of Prussian Blue Modified Electrodes. *J. Am. Chem. Soc.* **1982**, *104* (18), 4767–4772. https://doi.org/10.1021/ja00382a006.

(2)	McCargar, J. W.; Neff, V. D. Thermodynamics of Mixed-Valence Intercalation Reactions: The Electrochemical Reduction of Prussian Blue. *J. Phys. Chem.* **1988**, *92* (12), 3598–3604. https://doi.org/10.1021/j100323a055.

(3)	Hamer, W. J.; Wu, Y.-C. Osmotic Coefficients and Mean Activity Coefficients of Uni-Univalent. *J Phys Chem Ref Data* **1972**, *1*.
